# Biofouling-resistant nanomaterials for non-enzymatic glucose sensors: A critical review

**DOI:** 10.1016/j.mtbio.2025.101746

**Published:** 2025-04-08

**Authors:** Fareeha Arshad, Israr U. Hassan, Jwaher M. AlGhamadi, Gowhar A. Naikoo

**Affiliations:** a Department of Mathematics and Sciences, College of Arts and Applied Sciences, Dhofar University, PC 211, Salalah, Oman; b Department of Chemistry, College of Science, Imam Abdulrahman Bin Faisal University, Dammam, 31451, Saudi Arabia

**Keywords:** Antifouling, Biofouling, Nanomaterials, Glucose, NEGS, Sensors

## Abstract

Biofouling is a significant concern in sensors and diagnostic applications as it results in reduced sensitivity, selectivity, and response time, false signals or noise, and ultimately causes a reduction in the sensor lifespan. This is particularly a concern while developing non-enzymatic glucose sensors (NEGS) that can be used to fabricate implantable sensors for continuous glucose monitoring. Thus, developing advanced materials solutions in the form of nanomaterials that display inherent antifouling activity is imperative. Due to their small nanosized dimensions and tunable microstructures, nanomaterials display unique physio-chemical properties that display antifouling efficiency and thus can be applied towards developing highly stable, sensitive, and selective NEGS. Through this review, we aim to explore the recent advances in the field of antifouling nanomaterials that offer promising potential to be applied towards developing NEGS. We discuss the details of various biofouling-resistant nanomaterials, including graphene and graphene oxide, carbon nanotubes, gold nanoparticles, silver nanoparticles, metal oxide nanoparticles, and polymeric nanocomposites. Further, we highlighted the possible mechanism of action involving nanomaterials in providing antifouling features in NEGS, followed by a brief discussion of the advantages and disadvantages of using nanomaterials for antifouling in developing NEGS. Finally, we concluded the article by proposing the future prospects of this promising technology.

## Highlights

• Biofouling undermines sensors, ripping sensitivity, accuracy and life span.
• This review explores recent advances in antifouling nanomaterials for NEGS development.
• We highlight biofouling resistant nanomaterials, mechanisms, and their role in NEGS.
• Classification of nanomaterials with examples, highlighting pros and cons for antifouling.
• Further research is needed to expand the scope of novel antifouling nanomaterials.

## Graphical abstract

**Figure:**
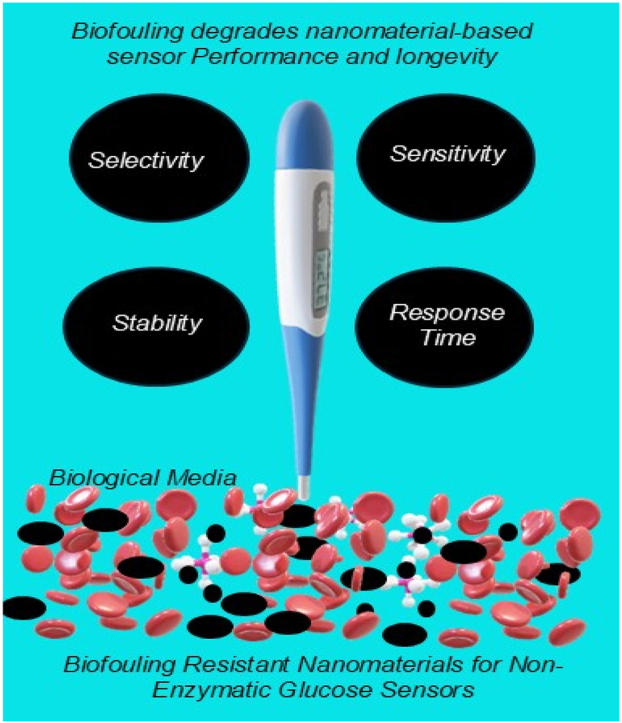

## 1 Introduction

Glucose management and monitoring are essential for diabetes management as diabetes mellitus is a constantly growing global health concern that has affected millions of people worldwide regardless of age or gender [1]. To achieve continuous glucose monitoring and diabetes management, traditional enzyme-based glucose sensors involving glucose oxidase have been commonly used owing to their high sensitivity and selectivity in detecting glucose molecules. However, because of the involvement of biological enzymes, such sensors face multiple disadvantages, including sensor instability, especially under varying environmental conditions like changing pH and temperature, and the involvement of complicated and expensive fabrication steps [2]. Therefore, the reliability and shelf-life of the enzymatic glucose sensor remain at stake. Thus, the previously mentioned disadvantages can be overcome with a shift towards non-enzymatic glucose sensors [3,4] (see , Fig. 5, Fig. 6, Fig. 7).

**Fig. 1:**
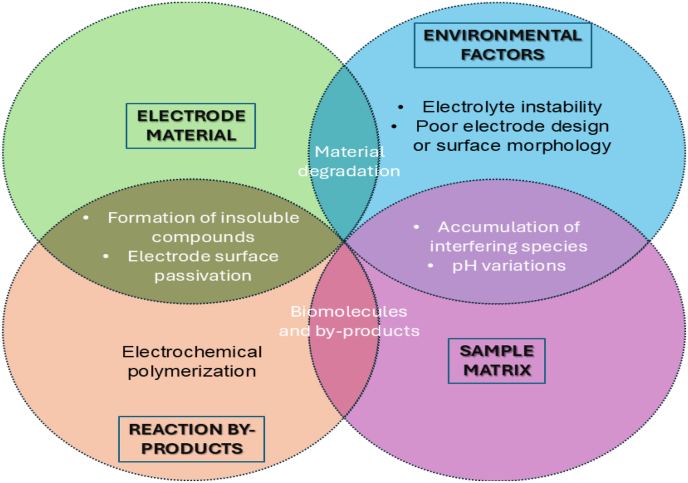
Venn diagram illustrating the factors contributing to electrode fouling in NEGS. The three primary categories are electrode material, environment, sample matrix, and reaction by-product-related factors. Overlapping areas indicate interactions between these categories that collectively contribute to sensor fouling.

Despite the promising prospect of non-enzymatic glucose sensors (NEGS), a critical challenge remains: biofouling. Biofouling is a process in which biological materials like biomolecules like proteins, peptides, carbohydrates, cells, and microorganisms accumulate over the sensor surface, decreasing the sensor performance, shelf life and sensitivity [5]. Accumulating such an impermeable layer over the electrode surface affects the analytical performance and increases background noise due to the electrode biofouling, which thus cannot detect significantly low signals. Biofouling is especially a concern in implantable glucose sensors as such devices are continuously exposed to biological fluids like whole blood or interstitial fluids rich in fouling agents [6]. Thus, it is imperative to incorporate biofouling resistance to ensure long-term functioning and accuracy of the NEGS. Electrochemical measurement on its own also helps in the desorption of species with the help of electrochemical pulses that decrease the adsorption of fouling agents and aid in removing the coating from the electrode surface [7]. Such an electrochemical cleaning process can remove commonly adsorbed species formed during oxidation-reduction reactions. However, the process could be harmful, especially when the surface is modified with biochemical enzymes or catalysts, resulting in their loss or physical removal from the sensor surface [8]. Multiple factors collectively contribute to electrode fouling, as displayed in Fig. 1.

**Fig. 2:**
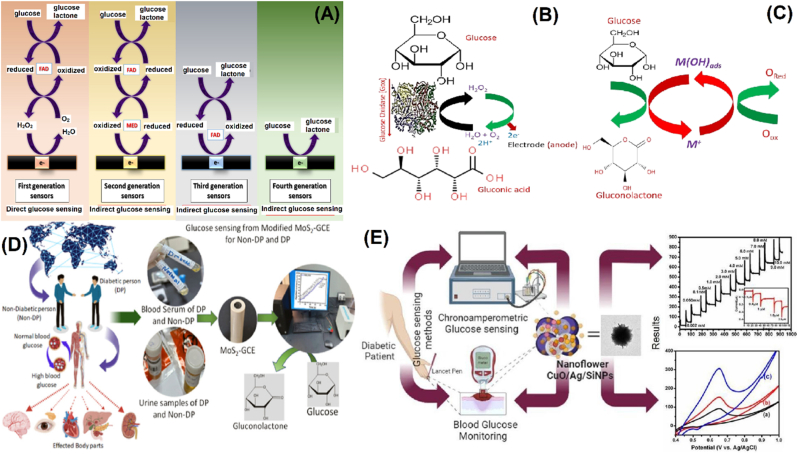
Glucose detection using non-enzymatic glucose sensors. (A) Mechanism of action followed during direct and indirect glucose detection using different glucose sensors. Adapted with permission from Ref. [34], copyright (2021) AiChE and ref. [35], copyright (2023) Elsevier. (B) In-vitro measurement of glucose concentration via amperometric method. (C) The incipient hydrous oxide adatom mediator (IHOAM) model. Adapted with permission from Ref. [36], copyright (2021) Elsevier and ref. [1], copyright (2023) Frontiers. Examples of NEGS. (D) Enzyme-free 2D-MoS_2_ nanostructures for ultra-sensitive glucose sensors with real-time health-monitoring capabilities. Adapted with permission from Ref. [37], copyright (2024) ACS. (E) Non-enzymatic glucose sensors comprise high-performing NFS-CuO/Ag/SiNPs-based composite material. Adapted with permission from Ref. [38], copyright (2023) Elsevier.

Nanomaterials have emerged as promising candidates to overcome biofouling in glucose sensing applications owing to their unique physicochemical properties, like enhanced surface area, rapid catalytic activity, and adjustable surface chemistry (see Fig. 2). Multiple studies in the recent decade have focused on exploring metallic nanomaterials like gold [9], platinum [10], and copper [11] nanoparticles that display enzyme-mimicking properties towards glucose oxidation in non-enzymatic biosensors. Such nanomaterials have been shown to function under various conditions, offering enhanced stability compared to their biological enzymes. To confer anti-fouling properties to such nanomaterials, they are often coated with a protective layer as a barrier to prevent contaminants from directly contacting the electrode surface [12]. Materials that are hydrophobic or that contain uncharged hydrogels prevent fouling via the barrier effect and exhibit enhanced repulsive hydration forces [13]. In addition, surface modification of nanomaterials with anti-fouling coatings like polyethene glycol (PEG) [14], zwitterionic polymers [15], and self-assembled monolayers (SAM) [16], the modified nanomaterials not only display enhanced glucose oxidation capability but also have shown to resist biofouling. PEG is not toxic, biocompatible, and can easily link to the electrode surface; therefore, it is promising for anti-fouling applications in glucose sensors [17]. Zwitterionic molecules and SAM, on the contrary, display enhanced oxidative resistance and hydrolytic stability [18]. Such coatings form hydrophilic layers that repel protein adsorption and cellular attachment, thereby maintaining the sensor performance in biological fluids [19]. Another strategy is using hybrid nanomaterials that combine metallic nanoparticles with other nanostructures like metal-organic frameworks [20] and carbon-based nanomaterials like graphene oxide [21] or carbon nanotubes [22] that display inherent resistance towards biofouling. Carbon nanostructures protect the sp^3^ hybridization effect by providing a larger surface area. Thus, such hybrid nanostructures display synergistic effects such that the carbon nanoparticle displays anti-fouling properties, and the metal nanoparticle in the hybrid nanostructure provides glucose oxidation capability. Other nanocomposites incorporating transition metal oxides like nickel [23] and cobalt oxides [24] have also shown excellent glucose oxidation capability with high sensor stability [25,26]. Functionalizing these nanostructures with anti-fouling agents can also aid in improving the sensor performance, especially under complex biological environments that can help prevent non-specific binding and biofouling.

Though several antifouling strategies have been explored to enhance the stability and performance of NEGS, challenges remain in achieving long-term efficiency and adaptability across diverse applications. These limitations highlight the need to comprehensively analyse emerging approaches and innovative solutions. In this review, we critically examine recent advancements in antifouling strategies, emphasizing novel material designs and integration techniques that push the boundaries of conventional methods. Thus, our work provides a structured perspective on future directions for optimizing NEGS performance. Though there have been a few review articles published in recent years on exploring different materials for anti-fouling, they have primarily focused on marine anti-fouling [27,28], anti-fouling for water treatment purposes [18,29], or general anti-fouling strategies for biosensing purposes [30,31]. However, to the best of our knowledge, no comprehensive literature discusses the application of biofouling-resistant nanomaterials for NEGS. Therefore, through this article, we aim to explore the promising potential of using nanomaterials to develop NEGS that do not display any biofouling. After a quick introduction to the topic, we have provided a detailed overview of the different types of biofouling-resistant nanomaterials that can be used for developing NEGS. Subsequently, we highlighted the possible mechanism of action of nanomaterials for biofouling-resistant action in NEGS. Finally, we concluded the article by discussing the possible challenges and future directions of this promising field.

## 2 Types of biofouling-resistant nanomaterials

As discussed in the previous section, the primary issue lies in applying the non-enzymatic glucose sensors, especially towards glucose detection in biological media, owing to biofouling of the electrode surface of the sensors resulting in a reduction in the sensor accuracy, stability, and selectivity. Multiple nanoparticles and their composites have been explored in the recent decade for their biofouling-resistant activities and potential application in non-enzymatic glucose sensors. This section will explore the prospects of graphene and graphene oxide, carbon nanotubes, gold nanoparticles, silver nanoparticles, metal oxide nanoparticles, and polymeric nanocomposite towards their biofouling-resistant properties.

### 2.1 Graphene and graphene oxide

Graphene is a single sheet carbon material comprising carbon atoms linked through strong sp^2^ hybridized sigma bonds, thus allowing a large surface area, flexibility, enhanced conductivity and mechanical strength and improving charge carrier properties. Graphene and its derivatives have also been shown to display promising applications like energy storage, surface coating, and filtration processes. Because of the hydrophobic nature, the graphene surface easily disallows the attachment of any biomolecule over the sensor surface, thereby preventing biofouling activities. Also, graphene structures are usually modified with other hydrophilic or amphiphilic functional groups to improve their anti-fouling applications. For instance, functional groups like –OH, –COOH, or –SO_3_H can modify the hydrophilicity and relevant applications of graphene. For instance, different functionalized graphene nanosheets were investigated upon incorporation with polyether sulfone membrane [32]. The authors observed that the membrane morphology remained unchanged with the inclusion of functionalized graphene; however, the hydrophilicity and antifouling capacity significantly improved. Similarly, in another study, graphene nanosheets were prepared using a less toxic cationic antimicrobial material that was then applied in developing poly-sulfone membranes that displayed remarkable antifouling properties against proteins like bovine serum albumin [33].

Graphene derivatives like graphene oxide (GO), reduced graphene oxide (rGO), or their functionalized counterparts already have inherent functional groups like hydroxyl, carboxyl, and epoxy groups over their surface that confer high dispersive capacity and prolonged stability. In particular, the hydrophilicity in GO molecules provides anti-adhesive features over the electrode surface, thereby reducing the deposition of fouling agents. This was observed in a recent study in which GO sheets were incorporated over a polyamide active layer over a narrow nanocomposite film, resulting in antifouling properties [39]. The study observed that the antifouling properties dramatically increased with an increase in GO loading over the film nanocomposite membrane. Another study showed the promising contribution of GO-based composite membranes with excellent hydrophilic and antifouling features [40]. A similar study used a unique interfacial polymerization technique to develop a GO-based thin film composite membrane with enhanced surface functionalities [41]. The presence of the hydrophilic GO reduced the deposition and adsorption of fouling agents over the membrane surface to enhance the antifouling properties. GO nanosheet stacking results in the formation of nanochannels that allow precise separation of foulants owing to interlayer spacing [42]. Moreover, the oxygen-rich groups available in GO confer high hydrophilicity and modification capacity for anti-fouling applications [43]. However, GO still has some drawbacks, like nanoparticle aggregation capacity and the ability to migrate, that influence hydrophilicity. Metal cations like Ca^2+^ tend to adsorb over the nanosheets with hydroxyl and carbonyl functional groups, potentially leading to GO aggregation [44]. Moreover, the 2D structure of GO can act as a physical barrier, restricting nanoparticle migration by immobilizing them within its network [45]. Functionalizing GO provides active sites with the dispersive and adsorptive capacity of multiple salts, broadening their application potential. Also, rGO, because they have a reduced number of functional groups compared to GO, provide sp^2^ structures and unique physical properties that confer superhydrophobic properties to the surface, thereby making them promising towards antifouling applications in biosensors. This is because the reduction of GO leads to the removal of many oxygen functional groups [46], thereby increasing the carbon content and contributing to the superhydrophobicity of the rGO [47]. The superhydrophobic nature of rGO prevents water from wetting the surface, causing the contaminants to slide off and resulting in a self-cleaning effect for the electrodes. This property is beneficial for preventing fouling in air-exposed environments or in surfaces where water repellency minimizes adhesion of pollutants. Thus, the choice between GO and rGO depends on the application and environmental conditions. Therefore, graphene and its derivatives can be potentially applied towards developing novel non-enzymatic glucose sensors.

### 2.2 Carbon nanotubes

Carbon nanotubes (CNTs) are hollow nanostructures with concentric cylindrical morphology consisting of one or more graphene sheets layered in concentric layers forming single or multiwalled carbon nanotubes (MWCNTs), respectively. CNTs, owing to their easy and inexpensive availability, physio-chemical properties like 1D tube structure, high conductivity, electrocatalytic properties, and enhanced surface area to volume ratio, serve as a very attractive electrode option for developing NEGS. Because of multiple delocalized π orbitals, CNTs allow easy linking with other materials through π-π stacking interactions [48]. Therefore, multiple studies have recently explored the prospects of applying CNTs towards developing highly sensitive and accurate NEGS. However, because of the poor dispersive properties of CNTs, especially under aqueous conditions because of their hydrophobic features, they result in agglomeration over the electrode surface, causing electrode fouling [49]. Usually, functional groups are used for CNT modification to enhance their hydrophilicity and improve their anti-fouling nature. For instance, in a recent study, hydroxyl and carboxyl functionalized MWCNTs were developed that displayed good anti-fouling properties [50].

Improvising MWCNT surface with hydrophilic functional groups like nitrogen or oxygen conferred further dispersive features to the MWCNTs. For instance, in a recent study, sulfonic acid functionalization of CNTs improves the hydrophilic properties of the nanomaterial along with providing dispersive features, agglomerative properties, and an increase in the overall antifouling nature of the nanomaterial. In addition, polymers have also been explored towards CNT modification to enhance the antifouling capacity and overall biocompatibility towards biomolecules. For instance, heparin-like macromolecules have functionalized CNTs with excellent antifouling nature [51]. Likewise, other polymeric materials like chitosan [52] and ionic liquids [53] have also been used towards MWCNTs functionalization to improve their antifouling properties. A few studies have also explored modifying MWCNTs surface with biomolecules like β-cyclodextrin [54] and BSA [55] to improve their antifouling capacity. The reduced hydrophilicity and enhanced stability of CNTs make them highly promising for developing anti-fouling surfaces. However, there are a few disadvantages that need to be addressed. For starters, the removal of aligned forests of CNTs requires harsh chemical etching methods. Further vigorous steps, such as plasma oxidation, are needed to open the ends of CNTs for their application as filtration media. Both steps add to the overall cost of CNT production and application and are also very tedious to optimize [56]. Thus, though CNTs display promising potential to serve as bio-fouling resistant nanomaterial towards developing highly sensitive and selective NEGS, further work is needed to reach the ultimate potential of CNTs for their superior application as antifoulants in NEGS.

### 2.3 Gold nanoparticles

**Fig. 3:**
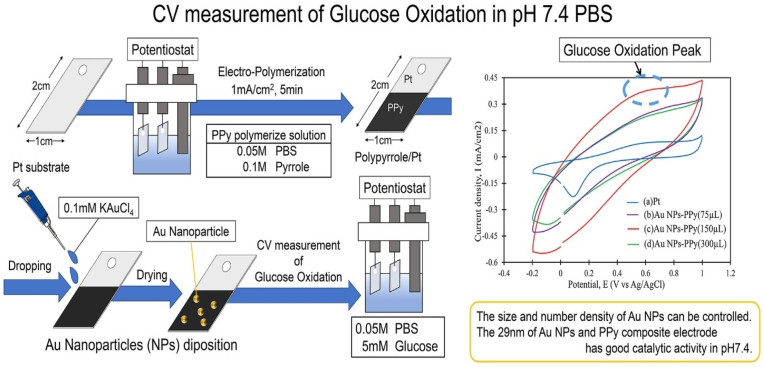
Mechanism of glucose detection with the help of polypyrrole/nano-gold composite-based NEGS. Reproduced with permission from Ref. [58], copyright (2022) Elsevier. (For interpretation of the references to colour in this figure legend, the reader is referred to the Web version of this article.)

**Fig. 4:**
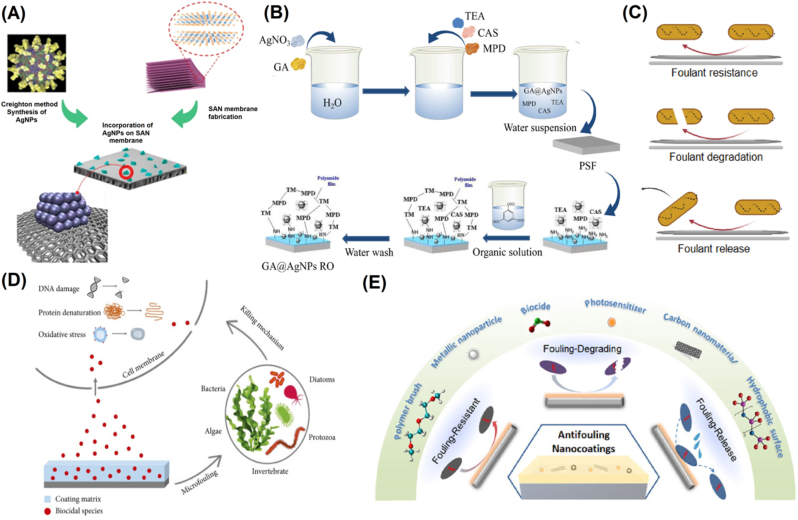
(A) Fabrication process of the AgNP-incorporated SAN membrane. Adapted with permission from Ref. [64], copyright (2023) Springer. (B) The process of preparation of the antifouling membrane. Adapted with permission from Ref. [65], copyright (2023) Degruyter. (C) Basic pathways involved towards anti-fouling activities displayed by AgNPs-based NEGS. (D) Schematic representation of possible antifouling mechanism. Adapted with permission from Ref. [66], copyright (2022) Wiley. (E) Schematic illustration of the principal strategies and active ingredients in coatings for antifouling. Adapted with permission from Ref. [28], copyright (2021) Frontiers.

Gold nanoparticles (AuNPs) have gained significant interest in developing NEGS due to their excellent electrical conductivity, large surface area, and biofouling-resistant properties. AuNPs possess unique physicochemical properties that enhance their role as non-enzymatic glucose sensors [57]. Their high surface-to-volume ratio allows for an increased number of active sites, which in turn boosts the sensitivity of the sensor to glucose molecules. In addition, AuNPs exhibit excellent conductivity, enabling more efficient electron transfer during glucose oxidation, which enhances the sensor's overall response time and detection accuracy [58], as shown in Fig. 3 Recently, multiple studies have been conducted to develop modified NEGS incorporating gold nanoparticles that display inherent antifouling properties [59]. For instance, a recent study developed an electrode coating consisting of a porous BSA matrix embedded with gold nanowires and glutaraldehyde [60]. The team recorded that the resultant nanostructure displayed enhanced antifouling properties and could retain the electrochemical response even on prolonged duration of exposure against complex biofluids, unlike the conventional antifouling layers used. In another similar study, l-cysteine functionalized poly(but-3-yn-1-yloxy)-2-oxo-1,3,2-dioxaphospholane as zwitterionic polyphosphoesters were used to functionalize AuNPs that displayed superior antifouling features [61] (see Fig. 4).

**Fig. 5:**
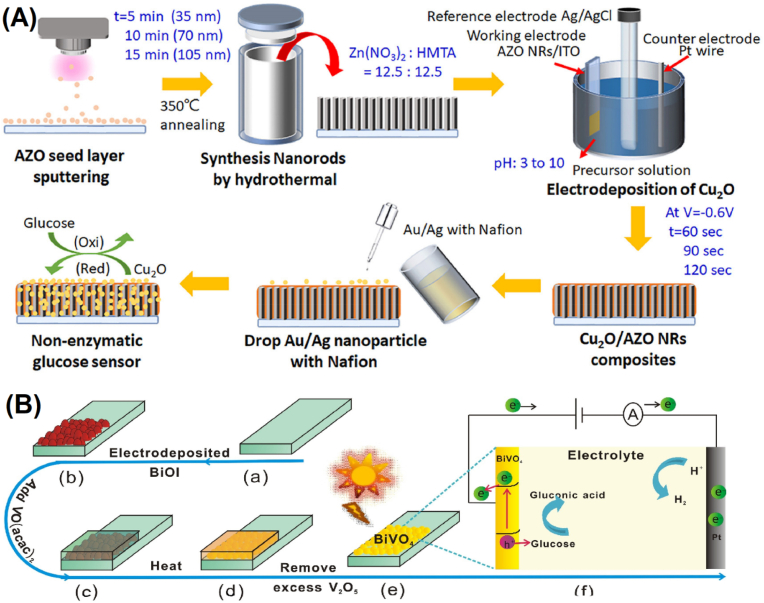
(A) The hybrid Au or Ag NPs/AZO NRs/Cu_2_O/ITO-based NEGS fabrication process. Adapted with permission from Ref. [75], copyright (2022) Elsevier. Synthesis process of BiVO_4_ sensor for glucose detection. Adapted with permission from Ref. [77] (2019) Elsevier.

**Fig. 6:**
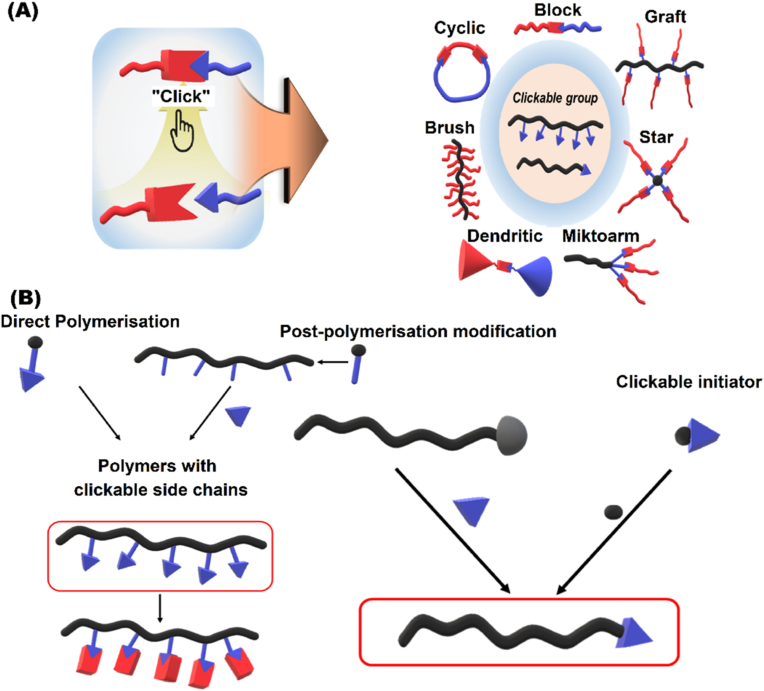
(A) Overview on the construction of macromolecules with various architectures through click coupling of functional polymers. Adapted with permission from Ref. [97], copyright (2021) Wiley. (B) Synthetic route for polymers with functional chain ends: (i) postpolymerization modification and (ii) polymerization using a clickable initiator. Adapted with permission from Ref. [97], copyright (2021) Wiley.

**Fig. 7:**
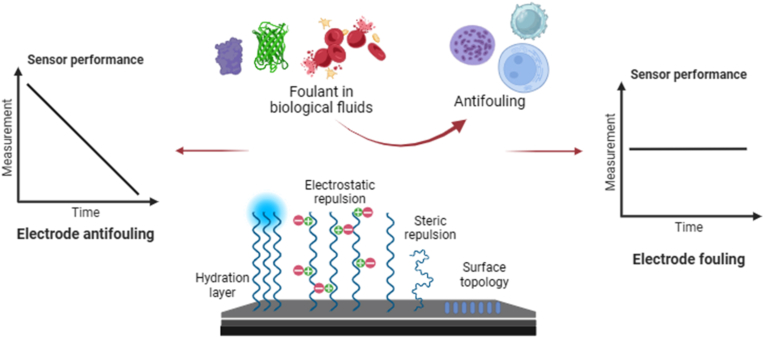
Different mechanism actions involved in providing electrode anti-fouling in NEGS with the help of nanomaterials.

Other studies have also explored the functionalization of AuNPs with distinct functional groups to improve their antifouling properties. For instance, hyperbranched polyester AuNPs were developed to be functionalized with carboxylic acids and linked to chitosan. The resultant nanoparticle-based electrode displayed excellent sensing activity and significantly reduced biofouling properties [62]. In addition, structures like L-cysteine betaine (Cys-b) have also been shown to add to the biofouling-resistant properties owing to their significantly active hydrophilic properties [63]. In the study, the group developed Ag@Au nanoshells modified Cys-b that displayed superior stability and improved antifouling properties. Therefore, AuNPs are promising to be applied towards developing antifouling nanomaterials for developing highly sensitive and selective NEGS.

### 2.4 Silver nanoparticles

Silver nanoparticles (AgNPs) are known for their high electrical conductivity and glucose oxidase-like properties. AgNPs are widely known for their excellent electrical conductivity and catalytic activity, essential for glucose sensing. The high surface area-to-volume ratio of AgNPs provides a large number of active sites for glucose molecules, further improving sensor performance. Additionally, the small size of AgNPs allows them to be dispersed uniformly over electrode surfaces, enabling efficient glucose oxidation at low applied potentials. As a result, AgNP-based non-enzymatic sensors exhibit high sensitivity and low detection limits, making them suitable for real-time glucose monitoring in clinical and wearable applications.

One of the significant advantages of AgNPs is their inherent biofouling resistance owing to their antimicrobial activity by generating reactive oxygen species and also because of the ability of AgNPs to create a low-adhesion surface, thus reducing the electrode affinity towards potential foulants [67]. Various recent works have been performed to study the applications of AgNPs for their antifouling properties towards biosensing applications. For instance, in a study by Mahmoudi and colleagues, GO-functionalized AgNPs were developed to display high antifouling properties [68,69]. Another study by Shafai and team explored hybrid material of AgNPs, zinc oxide, and titanium oxide nanoparticles that display inherent resistance against biofouling activities [70]. Similarly, other works have also investigated the prospects of Ag-modified graphitic carbon nitride [71] and Ti_3_C_2_T_X_ Mxene integrated with AgNPs-GO [72]. Incorporating carbon base nanostructures confers the resultant electrode support and electron-accepting properties that enhance the dispersive properties of Ag within the aqueous environment, suppressing the charge recombination of AgNPs [18]. Fan et al. also confirmed this in a study experimenting with applying Ag_3_PO_4_-GO as an antifouling agent [73]. With such a modification, the electrode nanostructure displayed excellent antifouling properties and improved permeation and rejection. The team observed enhanced removal efficiency against humic acids and other bacterial species like *E. coli* and *S. aureus*. However, the anti-fouling activity of AgNPs, like other nanoparticles, highly depends on their concentration, shape, size, and surface modifications [74]. Therefore, AgNPs can play a crucial role in developing NEGS owing to their inherent antifouling properties; however, further work is needed, especially concerning optimizing the physicochemical properties of the AgNPs to develop those nanoparticles that can potentially display the best inherent anti-fouling behaviour.

### 2.5 Metal oxide nanoparticles

Metal oxide nanoparticles like zinc oxide (ZnO) [75], titanium dioxide (TiO_2_) [76], aluminium oxide [75], bismuth oxide [77], copper oxide (CuO) [78], iron oxide [79], manganese oxide [1], and nickel oxide (NiO) [23] offer an enzyme-free, durable alternative with enhanced performance for glucose sensing in complex biological environments, owing to their multiple oxidation states and ability to facilitate rapid electron transfer during the electrochemical reaction. Their high surface area-to-volume ratio further amplifies the sensor's sensitivity by providing more active sites for glucose interaction. As a result, sensors based on metal oxide nanoparticles can achieve fast response times, high sensitivity, and low detection limits, which are critical for accurate glucose monitoring, especially in applications like diabetes management.

Another critical advantage of metal oxide nanoparticles is their inherent resistance to electrode fouling and super-hydrophilic features, owing to their unique surface chemistry and ability to generate reactive oxygen species (ROS) [18]. ROS can inhibit microbial growth and prevent the formation of biofilms on the sensor surface, thus maintaining sensor performance over prolonged use. In addition, metal oxide nanoparticles can resist fouling with the help of electrostatic interaction and van der Waals forces. Multiple studies have explored the application of metal oxide nanoparticles towards antifouling properties. For instance, in a study by Jeong and colleagues, ZnO NRs displayed promising anti-fouling properties [80] owing to their ability to release ROS and zinc ions. Similar observations on ZnO NPs were made in a study by Yong and colleagues [81] and by Holken and team [82] that confirmed antifouling properties because of their enhanced surface-to-volume ratio and super hydrophobic properties [83]. In another study by Shukla and colleagues, a hybrid material comprising polyphenylsulfone and ZnO NPs was developed that showed excellent antifouling features against soluble anionic compounds with a fouled flux recovery ratio of more than 90 % [84]. Similar observations have also been made for TiO_2_ as an antifouling material [85,86]. However, TiO_2_ also display agglomerative properties that hinder their practical applicability. This can be prevented using zwitterionic materials to stabilize the nanomaterial [87]. Likewise, iron oxides (Fe_2_O_3_ and Fe_3_O_4_) have also been shown to display inherent resistance to biofouling conditions [88] and, therefore, hold promising potential to be applied towards developing highly active and sensitive NEGS for continuous glucose monitoring.

### 2.6 Polymeric nanocomposite

Polymeric nanocomposites have emerged as highly promising biofouling-resistant nanomaterials for developing NEGS. They are composed of a polymer matrix that is reinforced with nanoparticles, such as carbon nanotubes [89], metal oxides [90], or metallic nanoparticles [35]. The combination of polymers and nanoparticles creates a synergistic effect, improving the overall conductivity, flexibility, and mechanical strength of the sensor material. This enhanced conductivity is essential in non-enzymatic glucose sensors, as it allows for efficient electron transfer during the glucose oxidation process, leading to faster response times and higher sensitivity. The flexibility of polymeric nanocomposites also allows for their integration into wearable or implantable devices, which require materials that can withstand mechanical stress while maintaining sensor performance. The polymer matrix can be engineered with anti-fouling properties by incorporating hydrophilic polymers, such as polyethylene glycol (PEG) [91] or zwitterionic polymers [92], which repel proteins and microorganisms, preventing them from adhering to the sensor surface. This reduces the risk of biofilm formation, ensuring that the sensor remains operational in complex biological environments, such as blood or interstitial fluid, for extended periods. Moreover, the nanoparticles embedded within the polymer matrix can also contribute to biofouling resistance.

Other polymeric nanomaterials like polypyrrole that function as an electroactive polymer have also been explored for their anti-fouling properties. These are particularly interesting owing to the abundant functional groups available over their surface, allowing for easy surface modification of different biomaterials and nanomaterials that add to the antifouling features. Recent studies have also explored the options of PANI nanofibers [93], polydopamine [94], and chitosan nanoparticles [95] to be applied as antifouling agents. They thus can also potentially be applied to develop highly active electrode surfaces in combination with other carbon-based or metallic nanoparticles to develop NEGS. Other polymeric compounds include polyzwitterions that possess poly (amino acid) backbone along with ethyldiamine moiety along with carboxylate in the side chains that helps in effectively functionalizing the nanomaterials that help in enhancing the antifouling properties of the nanomaterials [96].

Moreover, zwitterionic poly(α-amino acids) with rigid α-helical structures help form non-fouling surfaces via rapid self-assembly. In addition, antiparallel helical poly(γ-(2-(2-(2-methoxyethoxy)ethoxy)ethoxy)esteryl glutamates) (P(EG_3_Glu)s) also help enhance surface properties [98]. It has also been observed that zwitterionic-modified nanomaterials outperform PEG-modified ones in antifouling, offering promising applications due to their superior biocompatibility, stability, and ligand exchange capabilities [18]. Similarly, polymeric materials like polyamidoamine nanomaterials [99], nano curcumin [100], and supramolecular nanoribbons [101,102] can also be employed towards developing inherently biofouling resistant electrode surfaces for NEGS fabrication.

Table 1 below briefly highlights the different nanocomposites that display inherent antifouling properties and thus can be effectively applied and commercialized as highly sensitive and selective NEGS.

**Table 1:** Classification of different types of nanomaterials, along with examples, based on their respective advantages and disadvantages towards anti-fouling activity.

Nanomaterial category	Advantages	Disadvantages	Type of antifouling nanomaterial	Nanostructure	Antifouling moiety	Antifouling %	Ref.
**Non-metallic**	Display enhanced and specific surface area, excellent thermal and electrical conductivity, mechanical flexibility, strong mechanical integrity, and favourable biocompatibility.	Easily undergo agglomeration	Graphene and graphene oxide	Sulfonated graphene (SG) and graphene oxide (GO)	Pure polyethersulfone	119.7 and 71.3 using SG and GO respectively	[32]
				Guanidyl-functionalized graphene nanosheets	Polysulfone	77.4	[33]
				Graphene oxide nanosheets	Thin film nanocomposite	129.4	[41]
				Graphene oxide sheets	Thin film nanocomposite	97	[39]
			Carbon nanotubes	Hydroxyl and carboxyl functionalized multiwalled carbon nanotubes	PVDF	90.4	[50]
				Polymer brush functionalized carbon nanotubes	Polyethersulfone	95	[51]
				Chitosan-wrapped multiwalled carbon nanotube	PEBA thin film nanocomposite	98.7	[52]
				β-cyclodextrin functionalized MWCNTs	Polyethersulfone	92	[54]
**Metallic**	Show high hydrophilicity and resistance to protein adsorption	Display toxicity	Gold nanoparticles	Gold and zirconia nanoparticles	Multiwalled carbon nanotubes	–	[57]
				AuNPs	Polypyrrole	–	[58]
				Chitosan wrapped around Au nanoparticles	Hyperbranched polyester nanoparticles with carboxylic acid functional groups	–	[62]
				Ag@Au nanoshells	l-cysteine betaine	–	[63]
				AuNP	l-cysteine-functionalized poly(but-3-yn-1-yloxy)-2-oxo-1,3,2-dioxaphospholane	–	[61]
			Silver nanoparticles	AgNPs decorated on graphene oxide nanoplates	Polyamide 6,6 matrix	135	[68]
				Silver functionalized graphene oxide	Polyvinylidene fluoride	99.97	[69]
				AgNPs, ZnO NPs and TiO_2_NPs	Graphene oxide	–	[70]
				Silver-modified graphitic carbon nitride	Polyethersulfone	87.7	[71]
				Ag_3_PO_4_-GO	PVDF	85	[73]
			Metal oxide nanoparticles	TiO_2_ and AuNP	Poly-3-aminophenyl boronic acid	–	[76]
				Tetrapodal shaped ZnO	Polythiourethane	–	[82]
				ZnO NPs	Poly(vinylidene fluoride)	–	[83]
				ZnO NPs	Polyphenylsulfone	98	[84]
				TiO_2_	Polyethersulfone	96	[85]
				TiO_2_	Polysulfone	–	[86]
				TiO_2_ nanofibers	Polyethersulfone	94 ± 1	[87]
**Polymeric**	No toxicity, easily undergoes biodegradation, biocompatible, enhanced electrical conductivity, and high stability.	Lowered antibacterial activity	Polymeric nanocomposite	Furan-modified poly(styrene-alt-maleic anhydride)	Ag/CNTs	–	[89]
				Poly (ether-ether-sulfone) (PEES)/polyethylene glycol (PEG)	ZnO	97.23	[91]
				Polyaniline/polyethersulfone	Ti_3_C_2_T_x_ (MXene)	99	[93]
				Nano-curcumin	Polyethersulfone	–	[100]

## 3 Mechanism of action

Multiple mechanisms of action pathways can be followed to overcome electrode fouling with the help of nanomaterials. In this section, we outline different potential reaction pathways, each discussing the role of nanomaterials towards developing antifouling NEGS for sensitive and continuous monitoring of glucose molecules.

### 3.1 Electrode modification with hydrophilic coatings

Hydrophilic layering over the nanomaterial-modified electrode surface of NEGS can help overcome issues associated with biofouling by repelling the biomolecules that otherwise adhere to the sensor surface with time. As discussed earlier, polymers like PEG are the most used hydrophilic coatings for antifouling. However, PEG is often grafted over the nanomaterial surfaces, forming a hydration layer that also results in steric hindrance, preventing biomolecules from reaching the electrode surface [103]. In addition, the hydration layer also lowers the surface energy of the electrode surface, thereby lowering non-specific adsorption over the electrode surface.

Therefore, other polymeric substrates like zwitterionic polymers like poly (sulfobetaine methacrylate) (PSBMA) and poly (carboxybetaine methacrylate) (PCBMA) can be potentially applied towards developing inherently biofouling resistant nanomaterials for NEGS fabrication [104]. Zwitterionic materials possess positive and negative charges, creating a highly hydrated surface through electrostatically bound water molecules. This hydration layer acts as an effective barrier against biomolecule adhesion. Zwitterionic nanomaterial coatings have enhanced antifouling properties, providing greater stability and operational life.

### 3.2 Modification of surface charge on the nanomaterial

Surface charge on nanomaterials also plays a critical role in influencing the biomolecule adhesion over the electrode surface. Nanomaterials engineered to carry a specific charge over their surface can help repel the biomolecules available in the biofluids, thereby reducing the intensity of biofouling by repelling the same charge biomolecules away from the electrode surface [105]. For instance, when modified to carry a negative charge, nanomaterials like graphene oxide (GO) can allow for antifouling properties. Furthermore, the surface charge can be dynamically tuned by modifying nanomaterials with charged functional groups such as carboxylates (-COO^-^), sulfonates (-SO_3_^-^), or amines (-NH_3_^+^). This charge-based repulsion mechanism can significantly reduce fouling, improving the sensor's long-term stability and sensitivity.

### 3.3 Nanomaterials as physical surface barriers

When the electrode surface is coated with a nanomaterial layer, especially those that demonstrate a high degree of surface roughness or hierarchical nanostructures, they potentially provide antifouling features to the NEGS as a physical barrier to fouling agents. Studies have shown that nanostructured surfaces, such as vertically aligned carbon nanotubes (VACNTs) [106] or nanowire arrays [107], reduce the contact area between the sensor surface and fouling agents. Creating a surface with nanoscale features makes it more difficult for large biomolecules to adhere and form a stable fouling layer. Thus, materials like VACNTs can potentially create a forest-like structure on the electrode surface that restricts the access of biomolecules, allowing glucose molecules to diffuse through the gaps and reach the active sites for oxidation [106]. Similarly, other nanostructured surfaces based on metal oxides, such as nickel oxide (NiO) or copper oxide (CuO) nanowires, can also provide antifouling properties [[108], [109], [110]] by preventing the formation of a continuous fouling layer in addition to providing catalytic activity for glucose oxidation.

### 3.4 Chemical functionalization of the nanomaterial

As discussed in the previous sections, chemical functionalization of nanomaterials imparts antifouling properties by introducing specific molecular groups that repel biomolecules. For instance, thiol-based self-assembled monolayers (SAMs), when functionalized with PEG or zwitterionic groups over metallic nanomaterials like gold or silver nanoparticles, provide antifouling properties by forming a physical barrier and repelling the biomolecules from the electrode surface. Moreover, functionalization with glucose-recognition ligands, such as boronic acids [111], can enhance the selectivity of non-enzymatic glucose sensors while reducing non-specific adsorption. Boronic acids form reversible covalent bonds with glucose, allowing for selective glucose detection in complex biological fluids without significant interference from other species. This dual function of selectivity and antifouling makes chemically functionalized nanomaterials highly effective in NEGS.

## 4 Advantages and disadvantages of using nanomaterials for antifouling in NEGS

As discussed in the previous sections, nanomaterials can be successfully applied as antifoulants in developing NEGS for their distinct advantages, especially their inherent physicochemical properties. The large surface area to volume ratio of nanomaterials like nanowires and nanotubes offer an enhanced surface area for electrodes, thereby offering a larger number of active sites for glucose detection, improving selectivity and sensitivity, and reducing the impact of fouling. Moreover, certain nanomaterials like gold, platinum, or copper-based nanomaterials offer enzyme-mimicking properties for glucose oxidation, thereby reducing the dependence on enzymes, which in turn causes fouling [112,113]. More importantly, nanomaterials can be easily functionalized with antifouling ligands or coatings like PEG or zwitterionic polymers, which help resist the adsorption of biofouling agents like proteins and lipids [114]. In addition, nanomaterials also provide for rapid electron transfer owing to their high conductivity, which helps overcome the effects of fouling, thereby improving the signal transmission and stability of the NEGS.

Despite the promising inherent antifouling activities observed in multiple nanomaterials, a few limitations must be addressed. For instance, nanomaterials like carbon-based nanoparticles and related nanocomposites undergo aggregation, reducing their overall surface area and loss in antifouling and glucose oxidase mimicking activities, leading to inconsistent sensor performance [115]. Moreover, loss of activity and nanomaterial degradation become more prominent with time and thus affect the sensor performance over extended use. More importantly, even though nanomaterials are often functionalized to resist fouling, they may not completely prevent biofouling, especially in complex biological environments; proteins, lipids, and other species can still adsorb over the electrode surface. Therefore, strategies such as careful surface modification and better nanomaterial stabilization can be adapted to produce highly efficient nanomaterials with inherent anti-fouling properties to overcome these disadvantages.

## 5 Future directions and conclusion

In this paper, we have comprehensively discussed the research advances of developing nanomaterials that display inherent antifouling properties and can be applied towards developing highly sensitive and selective NEGS. An ideal fouling-resistant nanomaterial must be stable, easily and inexpensively available, reliable, and exhibit reduced or no toxic behaviour. In addition, to be applied in NEGS, they must display glucose oxidase-like activity for rapid glucose oxidation and sensitive and selective glucose detection. As discussed in the previous sections, non-metallic and polymer-based nanomaterials display lower antifouling features than metallic nanomaterials. However, metallic nanomaterials display higher toxicity than polymeric or non-metallic nanomaterials, making them unsuitable for signal amplification in continuous glucose monitoring or implantable NEGS applications. Therefore, hybrid nanomaterials comprising metallic and non-metallic nanomaterials and polymers can be applied as they simultaneously display enhanced chemical tunability and good antifouling properties. In addition, zwitterionic polymers have also attracted much attention owing to their easy availability, synthesis, large number of functional groups, and application. As discussed earlier, the fouling-resistant properties of the nanomaterials and the mechanisms involved primarily depend on the hydrophilic properties of the nanomaterials. Thus, further work must be done to broaden the research and application scopes towards developing novel antifouling nanomaterials.

For instance, more work is needed to develop novel nanomaterials for fabricating next-generation glucose sensors with superior mechanical and antifouling properties, including the methods for allowing uniform dispersion of nanomaterials to overcome nanomaterial aggregation and instability to obtain consistent sensor signals. Moreover, the nanomaterial concentration, toxicity, and sensor durability must be investigated to understand further the future applicability of such biofouling-resistant NEGS to develop highly stable, fouling-resistant, and toxicity-free implantable sensors. Furthermore, additional research should be conducted to optimize the cost of developing large-scale, highly stable, and easy-to-use anti-fouling nanomaterials for their application in NEGS. More importantly, though nanomaterials and related research have exponentially increased in recent years, their application in the commercial field remains understated. Because most of the studies have been conducted in laboratory settings alone, further steps must be taken to consider their application in clinical and commercial settings for large-scale applications. Finally, the antimicrobial and toxicity behaviour of the nanomaterials remains a challenge for developing nanomaterials with high applicability in developing NEGS, especially those concerning continuous glucose monitoring like in implantable sensors. Thus, it is imperative to develop new inherently fouling-resistant nanomaterials that can be applied toward developing NEGS for large-scale usage.

## CRediT authorship contribution statement

**Fareeha Arshad:** Writing – original draft, Methodology, Formal analysis, Data curation. **Israr U. Hassan:** Writing – review & editing, Visualization, Project administration. **Jwaher M. AlGhamadi:** Writing – review & editing, Supervision, Project administration, Conceptualization. **Gowhar A. Naikoo:** Writing – review & editing, Visualization, Supervision, Project administration, Methodology, Formal analysis, Data curation, Conceptualization.

## Declaration of competing interest

The authors declare that there are no known competing financial interests or personal relationships that could have appeared to influence the work reported in this paper.

## Data Availability

Data will be made available on request.
